# The Prevalence of Giardia Intestinalis in Dyspeptic and Diabetic Patients

**DOI:** 10.5402/2011/580793

**Published:** 2011-07-27

**Authors:** Gözde Derviş Hakim, Şafak Kızıltaş, Hilmi Çiftçi, Şafak Göktaş, İlyas Tuncer

**Affiliations:** ^1^Department of Internal Medicine, Göztepe Training and Research Hospital, Health Ministry, 34862 Istanbul, Turkey; ^2^Department of Gastroenterology, Göztepe Training and Research Hospital, Health Ministry, 34862 Istanbul, Turkey; ^3^Department of Infectious Diseases, Health Ministry, Göztepe Training and Research Hospital, 34862 Istanbul, Turkey

## Abstract

*Background and Aims*. We aimed to investigate the prevalence of Giardiasis in patients with dyspepsia and patients with diabetes mellitus. *Methods*. 400 patients and 100 healthy persons were included in this clinical prospective study. The number of patients in each group was equal, 200 dyspeptic and 200 diabetic, respectively. The antigen of G. lntestinalis was determined in the stool specimens by ELISA method. *Results*. The frequency of Giardiasis was 7% in dyspeptic and 15% in diabetic patients. There was no positive results in any of the healthy persons. There was a significant difference in prevalence rate of Giardiasis between patients with dyspepsia and diabetes mellitus (*P* < 0.05). *Conclusions*. These results revealed that the prevalence of Giardiasis in dyspepsia and with diabetes mellitus was high in our country. This is the first study investigating the prevalence of Giardiasis in diabetic patients. To investigate Giardiasis in diabetic patients, who have dyspepsia or not, may be a good approach for public health.

## 1. Introduction

Dyspepsia is an important health problem, which leads to significant impairments in health-related quality of life and has affected large populations worldwide. The prevalence rate of dyspepsia ranging from 7% to 60% were reported in different population-based studies [1–3]. 

 Diabetes mellitus contributes substantially to the global burden of disease, with an estimated 150 million people affected worldwide, and its prevalence is expected to double by 2025 [4]. There are 3 million people with diabetes mellitus in our country [4]. Also, it is fact that, diabetic patients have prone to infectious diseases by various mechanisms [5, 6]. Disturbances in cellular innate immunity play a role in the pathogenesis of the increased prevalence of infections in DM patients. A second important mechanism is the increased adherence of the microorganisms to diabetic cells. Furthermore, some microorganisms become more virulent in a high glucose environment [5, 6].

Giardia intestinalis, also termed G. Lamblia and G. duodenalis, is a flagellated protozoan initially described by Van Leeuwenhook in 1681. It is a protozoan which most identified from gastrointestinal tractus [7]. Transmission of Giardia occurs by fecal-oral rout [8, 9]. That is why it has been implicated in multiple outbreaks in high-risk groups. High-risk groups include infants, young children and immunocompromised patients. The prevalence of Giardiasis in industrialized world is 2–7% [10]. In developing countries, which have poor sanitation and hygienic conditions, reported that prevalence rate is 40% [11, 12]. Giardiasis typically occurs following the ingestion of water or foods contaminated with fecal material containing cysts, and the infectivity dose may be as low as 10 cysts. The infection can be asymptomatic or patients may present extraintestinal symptoms, such as fever, maculopapular rashes, pulmonary infiltrates, lymphadenopathy, polyarthritis, and urticaria. However, the most common symptoms are diarrhea, abdominal pain, bloating, flatulence, and weight loss resulting from malabsorption [13]. Most of these symptoms are similar with presenting in patients with dyspepsia. The diagnosis of Giardiasis has been classically on detection of cysts or trophozoites in stool and duodenal aspirates specimens by direct microscopic examination and duodenal endoscopic biopsy specimen by histologically. Recently proven, ELISA methods for the detection of Giardia-specific antigen in fecal specimens have 97.5% sensitivity and 99.5% specificity [14, 15]. 

Since Giardiasis is an etiologic cause of dyspepsia and immuncomopromised patients, such as diabetics have prone to Giardiasis, we hypothesized that determination of prevalence rate may be useful for public health. For this purpose, we designed this clinical prospective study.

## 2. Materials and Methods

This was a prospective, single-center study. A total of 200 dyspeptic patients (male/female: 54/146) and 200 diabetic patients (male/female: 79/121), who were admitted to Göztepe Training and Research Hospital, outpatient clinics of Gastroenterology and Internal Medicine between July 2008 and January 2009, were enrolled into the study. 100 healthy subjects (male/female: 26/74) without any gastrointestinal complaints served as controls. The diabetic patients were divided into subgroups: patients with dyspepsia (*n* = 69) and without dyspepsia (*n* = 131).

Written informed consent was obtained from all participants. The study protocol was approved by the Local Research Ethics Committee. Dyspepsia and diabetes mellitus was defined according to the Criteria of Rome III consensus report and American Diabetes Association, respectively.

The patients who have any gastrointestinal malignancy and immunosuppressive condition were excluded. Demographic characteristics of patients and healthy subjects are shown in Table 1. 

Age, sex, symptoms, findings of history, and physical examination were recorded. The determination of Giardia-specific antigen in stool specimens were done by ELISA method with Generic Assays GmbH kit (Ludwing-Erhard-Ring 3, 15827, Germany). 

All data were collected and analyzed with NCSS 2007 & PASS 2008 Statistical Software (Utah, USA). One way Annova test was used to compare the patient and control groups for the normally distributed parameters. Statistical significance between the groups was determined using the chi-square and Fisher's exact test. *P* value less than 0.05 was considered statistically significant.

## 3. Results

This study was performed with 400 patients (200 dyspeptic and 200 diabetic) and 100 healthy subjects between July 2008 and January 2009. Table 1 shows the demographic characteristics of the study groups.

The prevalence of Giardiasis was found 7% (14/200) in dyspeptic patients and 15% (30/200) in diabetic patients. There was no positive result of Giardia antigen in any of the healthy subjects. There is a significant difference in prevalence rate between the study groups and the control group (*P* < 0.05).

The frequency of Giardia antigen positivity was found 15.9% (11/69) in diabetic patients with dyspepsia and 14.5 (19/112) without dyspepsia. There was no statistical significant difference between the two groups (*P* > 0.05). There was a significant difference in terms of the prevalence of Giardiasis between diabetic and dyspeptic patients (*P* < 0.05). The prevalence of Giardiasis in patients with diabetes mellitus was higher than patients with dyspepsia.  Table 2 shows the distribution of prevalence rate in all study groups.  Table 3 shows the comparative prevalence rate in all study groups. 

There was no significant relationship between patient's symptoms and Giardia frequency (*P* > 0.05). The frequency of symptoms in patients with dyspepsia and diabetes mellitus are shown Tables 4 and 5, respectively. As it shown, diarrhea is not common symptom in each study group.  Table 6 shows the distribution of the symptoms according to Giardia antigen results in patients with dyspepsia. There was only slight significant difference in patients suffering from heartburn (*P* = 0.049).

## 4. Discussion

Giardiasis is a common infection in our country, such as worldwide. The prevalence rate was reported 2–7% as in developed and 40% in developing countries, respectively [10, 16]. The prevalence was higher in the countries that lack proper sanitation and hygienic conditions than other parts of the world. For this reason, we hypothesized that the prevalence was high in our country. Diabetes mellitus and dyspepsia were distinct clinical entity which affected a very large population worldwide. We investigated the prevalence rate of Giardiasis in dyspeptic and diabetic patients. This study is the first investigating the prevalence of Giardiasis in patients with diabetes mellitus. 

In 2006, Grazioli et al. reported that the prevalence of Giardiasis was 6.5% (9/137) in patients with dyspepsia and irritable bowel syndrome [17]. This prevalence rate has been reported 15.5% by Carr Jr. et al. [18]. In a study, reported from Egypt, which is an endemic area, the prevalence was found 44% (96/220) in patients with dyspepsia [19]. There are several reports from different countries and investigators [11, 20].

In our study, the prevalence rate of Giardiasis was determined 7% (14/200) in dyspeptic and 15% [30/200] in diabetic patients. Also, in 69 subjects of diabetics have dyspeptic symptoms, and prevalence rate of Giardiasis was found 15.9% (11/69) in this subgroup. There was no significant difference between diabetic patients with or without dyspepsia (*P* > 0.05). There was no positive results of Giardia antigen in any of the healthy subjects. This finding has been provided a good evidence for positive correlation between dyspepsia and Giardiasis.

In previous studies reported that dyspeptic symptoms (e.g., abdominal pain and discomfort) are more common than diarrhea in patients with Giardiasis [17, 19]. Our results supported this finding. In our study, patients with Giardiasis more commonly presented with dyspeptic symptoms than diarrhea (Table 6). Therefore, an important finding of this prospective study is that there was no relationship between dyspeptic symptoms and Giardiasis. 

Our results suggested that the frequency of Giardiasis in dyspepsia was lower than that reported from endemic country. A slight increase was observed when compared to the results in western populations. There was no published research to compare the prevalence of Giardiasis in diabetic patients observed in this study. 

In conclusion, the prevalence of Giardiasis is high in patients with dyspepsia and diabetes mellitus. To investigate the causes of dyspepsia, it should be considered the Giardiasis as an etiologic factor. This approach will be useful in diagnosis and management of dyspepsia.Taking into consideration the high frequency of Giardiasis in diabetic patients with or without dyspepsia, we speculate that searching Giardiasis will be a good approach for public health.

## Figures and Tables

**Table 1:** The demographic characteristics of study groups.

	The patients with dyspepsia	The patients with D. mellitus	Healthy controls
Age (mean ± SD)	41.47 ± 13.48	54.45 ± 11.92	46.51 ± 15.74
Total number (*n*)	200	200	100
Female sex (*n*%)	146 (73%)	121 (60.5%)	74 (74%)
Male sex (*n*%)	54 (27%)	79 (39.5%)	26 (26%)

**Table 2:** The distribution of prevalence rate in all study groups. *P* < 0.05 as considered statistical significance.

	Giardia antigen positive (*n*%)	Giardia antigen negative (*n*%)	*P* value (compared with controls)
Dyspepsia	14 (7%)	186 (93%)	0.007*
Diabetes mellitus	30 (15%)	170 (85%)	0.001*
Diabetes mellitus with dyspepsia	11 (15.9%)	58 (84.1%)	0.001*
Diabetes mellitus without dyspepsia	19 (14.5%)	112 (85.5%)	0.001*
Healthy control	0 (0%)	100 (100%)	

**Table 3:** The distribution of the prevalence rate of Giardiasis and comparative significance level in all study groups. *P < 0.05, **P < 0.01. D. M = D. Mellitus.

	Giardia antigen (+) (*n*%)	Giardia antigen (–) (*n*%)	*P*
Dyspepsia	14 (7%)	186 (93%)	0.011*
D. mellitus	30 (15%)	170 (85%)	
Dyspepsia	14 (7%)	186 (93%)	0.007**
Control	0 (0%)	100 (100%)	
Dyspepsia	14 (7%)	186 (93%)	0.027*
D. M + dyspepsia (+)	11 (15.9%)	58 (84.1%)	
Dyspepsia	14 (7%)	186 (93%)	0.026*
D. M + dyspepsia (−)	19 (14.5%)	112 (85.5%)	
D. mellitus	30 (15%)	170 (85%)	0.001**
Control	0 (0%)	100 (100%)	
D. mellitus	30 (15%)	170 (85%)	0.851
D. M + dyspepsia (+)	11 (15.9%)	58 (84.1%)	
D. mellitus	30 (15%)	170 (85%)	0.901
D. M + dyspepsia (−)	19 (14.5%)	112 (85.5%)	
Control	0 (0%)	100 (100%)	0.001**
D. M + dyspepsia (+)	11 (15.9%)	58 (84.1%)	
Control	0 (0%)	100 (100%)	0.001**
D. M + dyspepsia (−)	19 (14.5%)	112 (85.5%)	

**Table 4:** Distribution of the symptoms of patients with dyspepsia.

Symptoms	*n* (number)	% (percentage)
Bloating	172	86
Abdominal pain	142	71
Heartburn	121	60.5
Abdominal discomfort	97	48.5
Abdominal fullness	70	35
Nausea	50	25
Early satiety	42	21
Constipation	34	17
Diarrhea	19	9.5

**Table 5:** Distribution of the symptoms in diabetic patients with dyspepsia.

Symptoms	*n* (number)	% (percentage)
Bloating	59	29.5
Heartburn	59	29.5
Abdominal pain	44	22
Abdominal discomfort	34	17
Abdominal fullness	26	13
Early satiety	16	8
Constipation	12	6
Nausea	5	2.5
Diarrhea	4	2

**Table 6:** Distribution of the symptoms according to Giardia antigen results in patients with dyspepsia.

Symptoms	Giardia antigen (+) *n* (%)	Giardia antigen (−) *n* (%)	*P* value
Bloating	14 (100%)	158 (84.9%)	0.117
Abdominal pain	8 (57.1%)	134 (72%)	0.236
Abdominal discomfort	5 (35.7%)	92 (49.5%)	0.321
Nausea	5 (35.7%)	45 (24.2%)	0.337
Heartburn	5 (35.7%)	116 (62.4%)	0.049*
Constipation	3 (21.4%)	31 (16.7%)	0.647
Abdominal fullness	2 (14.3%)	68 (36.6%)	0.092
Early satiety	1 (7.1%)	41 (22%)	0.187
Diarrhea	1 (7.1%)	18 (9.7%)	0.755

*Fisher's exact test: *P* < 0.05.
